# A Bio-Guided Fractionation to Assess the Inhibitory Activity of *Calendula officinalis* L. on the NF-*κ*B Driven Transcription in Human Gastric Epithelial Cells

**DOI:** 10.1155/2015/727342

**Published:** 2015-09-28

**Authors:** Elisa Colombo, Enrico Sangiovanni, Michele D'Ambrosio, Enrica Bosisio, Alexandru Ciocarlan, Marco Fumagalli, Antonio Guerriero, Petru Harghel, Mario Dell'Agli

**Affiliations:** ^1^Department of Pharmacological and Biomolecular Sciences, Università degli Studi di Milano, Via Balzaretti 9, 20133 Milan, Italy; ^2^Laboratory of Bioorganic Chemistry, Department of Physics, Università degli Studi di Trento, Via Sommarive 14, 38123 Trento, Italy

## Abstract

*Calendula officinalis* L. has been largely known for its topical anti-inflammatory properties; however, there are no experimental evidences about its antiphlogistic effect at the gastric level. To investigate whether marigold might exert an activity against gastric inflammation, a CH_2_Cl_2_ extract obtained from* C. officinalis* flowers was evaluated *in vitro* on the NF-*κ*B pathway. The lipophilic extract demonstrated a significant inhibitory effect on the NF-*κ*B driven transcription. The identification of active compounds was conducted by a bio-guided fractionation of the extract that afforded 16 fractions. Fraction J exhibited a concentration-dependent inhibitory activity on the NF-*κ*B driven transcription and significantly contributed to the antiphlogistic effect showed by CH_2_Cl_2_ extract. The main components of fraction J were loliolide and the fucoside acetates of *β*-eudesmol and viridiflorol. HPLC analysis of fractions D and E led to the identification and isolation of triterpene esters that showed a concentration-dependent inhibition of the NF-*κ*B driven transcription, with faradiol-3-myristate and the corresponding aglycone being the most active compounds. The present study provides some experimental evidences that* Calendula officinalis* L. may exert an anti-inflammatory activity on the gastric district by the inhibition of the NF-*κ*B system, identifying the compounds responsible, at least in part, for the observed effect.

## 1. Introduction

*Calendula officinalis* L. (marigold, Asteraceae) is an annual herb of Mediterranean origin, widely cultivated as an ornamental plant. Dried flowers' heads are characterized by the presence of steroids, terpenoids, free and esterified triterpenic alcohols (faradiol, arnidiol, and calenduladiol, mainly as myristate), phenolic acids, flavonoids (quercetin, rutin, narcissin, isorhamnetin, kaempferol), carotenoids, and other minor compounds [1]. Marigold apolar extracts have been largely applied for external use in the treatment of skin ulcerations, eczema, and conjunctivitis [2]. Several studies, both* in vitro* and* in vivo*, demonstrated (1) the biological properties of preparations from marigold flowers as antioedematous agent [3–5], (2) the activity in wound healing [6–8], and (3) their antioxidant properties, at both topical [9–12] and systemic level [13–15]. Among secondary metabolites, the flavonoids, the carotenoids, and the triterpene fatty acid esters seem to be the major responsible for the anti-inflammatory activity of* Calendula officinalis* [3–5, 16, 17].

Gastritis is one of the most common inflammatory diseases, affecting about 50% of the world's population. Gastric mucosa is continuously exposed to many noxious factors and substances that can alter its integrity and lead to inflammation. Among those factors, the infection by* Helicobacter pylori* (*H. pylori*) is the most relevant one.* H. pylori* is a Gram-negative pathogen that colonizes the stomach of humans and primates and is classified as a carcinogen type I. Usually acquired during childhood, the infection can persist in the gastric district causing chronic gastritis or evolving into more severe diseases, such as atrophic gastritis, peptic ulcer, or gastric adenocarcinoma [18]. As a consequence of untreated infections, the gastric epithelial cells secrete many cytokines and chemokines that are attracted to the mucosal layer neutrophils, lymphocytes, and macrophages responsible for the maintenance of the inflammatory status. Several studies indicate that nuclear factor *κ*B (NF-*κ*B) plays a crucial role at the molecular level of this process [19–21].

Aqueous preparations from marigold flowers have been traditionally used for the treatment of gastrointestinal diseases, such as gastritis, ulcers, and colitis. Two studies demonstrated the efficacy of herbal mixtures containing marigold in the therapy of duodenal ulcers, gastroduodenitis, and chronic hyposecretory gastritis [22, 23] and a more recent one described the hypoglycemic and gastroprotective activities of* Calendula officinalis* L.* in vivo* [24]. Moreover, another work showed the effect of marigold preparations in ameliorating inflammatory symptoms in an animal model of ulcerative colitis [25]. So far, no one has established the clinical effectiveness of* Calendula officinalis* L. extracts as anti-inflammatory agent by internal use [26]. Thus, the aim of this work was (i) to evaluate* in vitro* the anti-inflammatory activity of a CH_2_Cl_2_ extract from marigold flowers in a cellular model of gastritis and (ii) to identify mixtures and compounds responsible for this effect by a bio-guided fractionation of the CH_2_Cl_2_ extract, considering the NF-*κ*B system as the molecular target.

## 2. Materials and Methods

### 2.1. Reagents

Dulbecco's modified Eagle's medium (DMEM)/F12 (1 : 1), penicillin, streptomycin, L-glutamine, and trypsin-EDTA were from Gibco (Life Technologies Italia, Monza, Italy). Foetal bovine serum (FBS) and disposable material for cell culture were purchased by Euroclone (Euroclone S.p.A., Pero, Milan, Italy). Parthenolide, dimethyl sulfoxide (DMSO), and all solvents (from Sigma-Aldrich, Milan, Italy) were of high-performance liquid chromatography purity grade (>98%). Tumor necrosis factor alpha (TNF-*α*) was from ImmunoTools (Friesoythe, Germany). Human adenocarcinoma cells (AGS) were purchased from European Collection of Cell Culture (EACC, UK, cat number 89090402). NF-*κ*B-luc plasmid was a kind gift from Professor N. Marx (Department of Internal Medicine II-Cardiology, Ulm, Germany). Britelite plus was from PerkinElmer (Monza, Italy).

Flash chromatography (FC) was performed by Merck Kieselgel 60 (70 230 mesh), Merck RP 18 LiChroprep (40–65 *μ*m); TLC by Merck Kieselgel 60 PF254; high pressure liquid chromatography (HPLC) by Merck Hitachi L7100 pump, L7400 UV detector, D7500 integrator Rheodyne injector; HPLC column and method by Synergi Hydro column (150 × 10 mm, 4 *μ*m particle size, 80 Å pore size; Phenomenex, Torrance, CA, USA), 5.0 mL/min, *λ* = 210 nm. The eluents were acetonitrile (ACN) (A) and ACN/water 50 : 50 v/v (B); in method 1 the gradient was changed linearly from 0% to 85% A in 45 min; in method 2 the eluent A was applied in the gradient of 0% at *t* = 0, 37% at *t* = 30, 85% at *t* = 31, 85% at *t* = 45, and 100% at *t* = 46 min. Optical rotations were measured on a Bellingham + Stanley ADP 440 polarimeter. NMR was performed by Bruker Avance 400 (^1^H at 400 MHz, ^13^C at 100 MHz), 5 mm BBI probe, *δ* in ppm using residual solvent signals as internal reference (CDCl_3_ = 77.0, CHCl_3_ = 7.26 and CD_2_HOD = 3.31), *J* values in Hz, multiplicities, and peak assignments from ^1^H, ^1^H COSY, ^1^ *J*_CH_ (HSQC), ^*n*^ *J*_CH_ (HMBC), and NOESY experiments. NOESY data are reported as correlation map(s) between protons ^1^H↔^1^H; HMBC data are reported as (^13^C) → correlated to ^1^H. MS was performed by Bruker Esquire_LC multiple ion trap. Electrospray ionization (ESI) was as follows: positive ion mode, capillary voltage 4000 V, nebulizing pressure 30.0 psi, drying gas flow 7 mL/min, and temperature 300°C. Electron impact mass spectra (EIMS, HREIMS) were recorded on a Kratos MS80 spectrometer with home-built data system and electron ionization at 70 eV,* m/z* (rel.%).

### 2.2. Plant Material

Dry flowers of* Calendula officinalis* L. (variety Calypso Orange Florensis) were obtained and extracted as previously described [27, 28]. A voucher specimen (number 20040929) is deposited at the Laboratory of Bioorganic Chemistry (Trento, Italy).

### 2.3. Cell Culture

AGS cells (EACC number 89090402) were grown in DMEM/F-12 medium supplemented with 100 units penicillin/mL, 100 mg streptomycin/mL, 2 mM L-glutamine, and 10% heat-inactivated foetal bovine serum. The cells were incubated at 37°C in humidified atmosphere with 5% CO_2_ until confluence. For the NF-*κ*B driven transcription assay, AGS cells were plated at the concentration of 1.5 × 10^4^ cells/mL in 24-well plates with complete medium to reach about 80% of confluence. After 48 h, medium was replaced with FBS-free medium and cells were exposed for 24 h to the compounds or fractions under study in the presence of TNF-*α* (10 ng/mL) as proinflammatory stimulus. To study the NF-*κ*B nuclear translocation, cells were plated in 10 mm dishes (3 × 10^6^ cells/dish) with fresh complete medium for 48 h. Then, medium was replaced with FBS-free medium containing increasing concentrations of fractions in the presence of TNF-*α* (10 ng/mL) for 1 h. We previously found that the fractions H and I are highly cytotoxic to AGS cells [28], so these fractions were excluded from the biological assays.

### 2.4. Transient Transfection and Luciferase Reporter Assay

To assess the effect of different fractions and isolated compounds on the NF-*κ*B driven transcription, AGS cells were transiently transfected by the calcium-phosphate method with a plasmid containing the luciferase reporter gene under the control of the E-selectin promoter with three NF-*κ*B binding sites. Sixteen hours after transfection, cells were stimulated with TNF-*α* (10 ng/mL), in the presence of CH_2_Cl_2_ extract (1–20 *μ*g/mL) or fractions A–P and subfractions (0.05–20 *μ*g/mL) or individual compounds (1–50 *μ*M). After 24 h, cells were harvested and luciferase activity was measured using Britelite plus reagent on a luminometer. Parthenolide (final concentration 10 *μ*M) is a natural inhibitor of the NF-*κ*B driven transcription [29]; it was used as positive control and showed around 50% inhibition in all the experiments reported in the present study. The NF-*κ*B driven transcription activation was expressed as a percentage relative to TNF-*α* treatment alone (100%).

### 2.5. NF-*κ*B Nuclear Translocation

To clarify the effect of marigold fractions on the NF-*κ*B pathway, we focused on the nuclear translocation as well; AGS cells were stimulated with TNF-*α* (10 ng/mL) in the presence or in the absence of the CH_2_Cl_2_ extract (2.5–10 *μ*g/mL) or fraction J (2.5–7.5 *μ*g/mL) for 1 h. Nuclear and cytoplasmatic extracts were obtained with Nuclear Extraction Kit (Cayman Chemical Company, Ann Arbor, MI, USA) following manufacturer instructions. Briefly, cells were first pelleted and resuspended in ice-cold hypotonic buffer. Addition of detergent (NP-40 10%) broke the cell membranes allowing access to the cytoplasmatic fraction while maintaining the integrity of the nuclear membrane. After separation of the cytoplasmatic fraction, the pelleted nuclei were lysed in ice-cold extraction buffer, obtaining the nuclear fraction. Total protein concentration of the nuclear extracts was determined by the Bradford method [30]. 10 *μ*g of total nuclear extracts for each sample was used for the measurement of nuclear NF-*κ*B (p65 subunit) using a commercial ELISA kit (Cayman Chemical Company, Ann Arbor, MI, USA). The NF-*κ*B nuclear quantity was expressed as a percentage relative to TNF-*α* treatment alone (100%).

### 2.6. Statistical Analysis

Results represented the mean ± s.d. of at least three independent experiments replicated in duplicates or triplicates. Statistical analysis was performed with GraphPad Prism 6.0 software, using one-way ANOVA analysis of variance followed by Bonferroni's post-hoc test. The significance was set at *P* < 0.05.

## 3. Results and Discussion

### 3.1. Effect of Marigold CH_2_Cl_2_ Extract on the NF-*κ*B Pathway

*Calendula officinalis* L. is a botanical worldwide known for its topical anti-inflammatory properties that are supported by clinical studies. Oral use of marigold infusions for the treatment of gastrointestinal diseases, such as gastritis, is only acknowledged in traditional medicine and is not currently supported by clinical trials [26]. Among the components of marigold flowers, triterpene fatty acid esters, present in lipophilic extracts, are the main responsible for the anti-inflammatory effects [3, 4, 16, 17]. However, internal use of lipophilic preparations from marigold flowers is not recommended for their renowned toxicity. In fact, our group has recently succeeded in identifying fractions and pure compounds which occur in the lipophilic extract from* Calendula officinalis* flowers and are toxic at the gastric level [28]. In the same work we demonstrated that the composition of lipophilic extract was not altered by gastric digestion. Accordingly, two studies were performed both* in vivo* [5] and* ex vivo* [31] and revealed that marigold extracts could exert the anti-inflammatory effect even after oral administration. In fact, the consumption of extracts from marigold flowers led to a decrease in plasmatic proinflammatory cytokines (TNF-*α* and IL-1*β*), indicating a stability of the anti-inflammatory components through stomach passage [5, 31].

**Figure 1:**
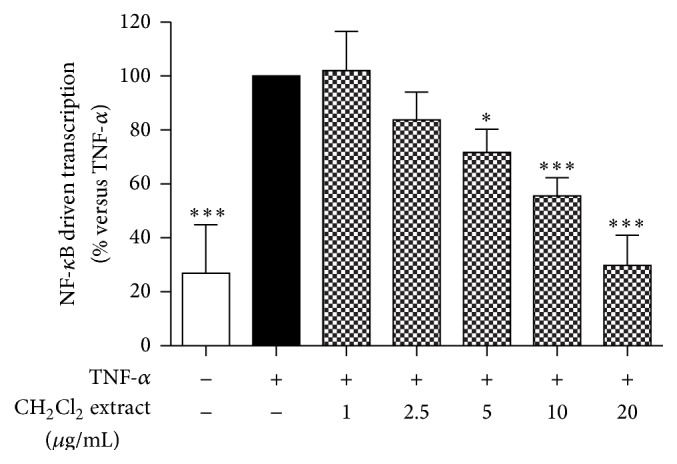
Effect of CH_2_Cl_2_ extract on the NF-*κ*B driven transcription. AGS cells were transiently transfected with NF-*κ*B-luc plasmid and treated with TNF-*α* in the presence of dichloromethane extract (1–20 *μ*g/mL) for 24 h. The NF-*κ*B driven transcription was proportional to luciferase signal measured. ^*∗*^ *P* < 0.05, ^*∗∗*^ *P* < 0.01, and ^*∗∗∗*^ *P* < 0.001.

In order to investigate if apolar constituents of marigold could exert anti-inflammatory effects at the gastric levels via inhibition of the NF-*κ*B pathway, the CH_2_Cl_2_ extract was assayed in a widely used human gastric epithelial cell line. AGS cells were transfected with a reporter plasmid containing the luciferase gene under the control of three NF-*κ*B responsive elements. Cells were then incubated with the extract in the presence of TNF-*α* as proinflammatory stimulus for 24 h. Luciferase activity was directly proportional to activation of the NF-*κ*B driven transcription. TNF-*α* alone increased luciferase activity by about fivefold when compared with the control cells. Dichloromethane extract obtained from* Calendula officinalis* L. flower heads inhibited the NF-*κ*B driven transcription in a concentration-dependent manner (IC_50_ of 6.31 ± 0.68 *μ*g/mL, mean ± s.d.), exhibiting a complete inhibition of TNF-*α*-induced NF-*κ*B activation at 20 *μ*g/mL (Figure 1).

When NF-*κ*B is activated by proinflammatory stimuli, it translocates into the nucleus, where it promotes the transcription of responsive genes, encoding for many mediators of the gastric phlogistic process. To clarify if CH_2_Cl_2_ extract could inhibit NF-*κ*B nuclear translocation as well, AGS cells were treated with TNF-*α* (10 ng/mL) as proinflammatory stimulus in the presence of the extract (5–10 *μ*g/mL) for 1 h. Cells were then lysed and nuclear fraction was separated; the nuclear NF-*κ*B was quantified through a commercial ELISA kit. Surprisingly CH_2_Cl_2_ extract did not exert a significant inhibitory effect on the NF-*κ*B nuclear translocation, thus suggesting that different mechanisms of the NF-*κ*B activation cascade might be involved.

### 3.2. Bio-Guided Fractionation of CH_2_Cl_2_ Extract and Characterization of the Fractions

The identification of biologically active compounds occurring in the lipophilic extract was achieved through a bio-guided fractionation. Briefly, a portion of the CH_2_Cl_2_ extract was subjected to FC on silica, increasing gradually the eluent polarity from hexane/EtOAc 95 : 5 to absolute EtOAc, and then washed with acetone. This procedure afforded 16 fractions (A–P); the triterpene esters were contained in fractions D-E (Table 1). All the fractions were assayed on AGS cells for their cytotoxicity, as previously described [28]. Nontoxic fractions were tested for their biological activity on the NF-*κ*B driven transcription in AGS cells stimulated with TNF-*α* for 24 h.

As shown in Table 1, 14 fractions were assayed but only 7 inhibited the NF-*κ*B driven transcription in a concentration-dependent manner. Fraction D showed only a mild biological activity, while the most active fractions were J and O, with an IC_50_ of 4.29 ± 0.97 *μ*g/mL and 7.82 ± 2.13 *μ*g/mL, respectively. Fraction J exhibited an inhibitory activity on the NF-*κ*B driven transcription comparable to that showed by the CH_2_Cl_2_ extract. Consequently, it was also assayed on the NF-*κ*B nuclear translocation, using the same model described above. Fraction J (2.5–7.5 *μ*g/mL) did not inhibit the NF-*κ*B nuclear translocation, thus confirming that both the extract and fraction J could directly prevent the activation of NF-*κ*B driven transcription. Namely, natural products present in the CH_2_Cl_2_ extract and in fraction J could exert their inhibitory effect on the binding of NF-*κ*B to DNA. This mechanism of action has already been demonstrated for other natural compounds, such as the sesquiterpene lactone parthenolide [32] and artemisinin [33, 34].

#### 3.2.1. Biological Activity of Triterpene Esters Present in Fractions D-E

All the biologically active fractions were analysed in RP-HPLC to identify the components responsible for the inhibitory effect on the NF-*κ*B driven transcription. The analysis was firstly devoted to the identification and isolation of triterpene esters present in marigold flowers that have been previously described for their anti-inflammatory properties [3, 4, 16, 17]. Thus, myristic esters of faradiol, arnidiol, and calenduladiol were identified and isolated in fractions D and E by HPLC analysis, as previously described [27], and then assayed for their biological activity.

**Figure 2:**
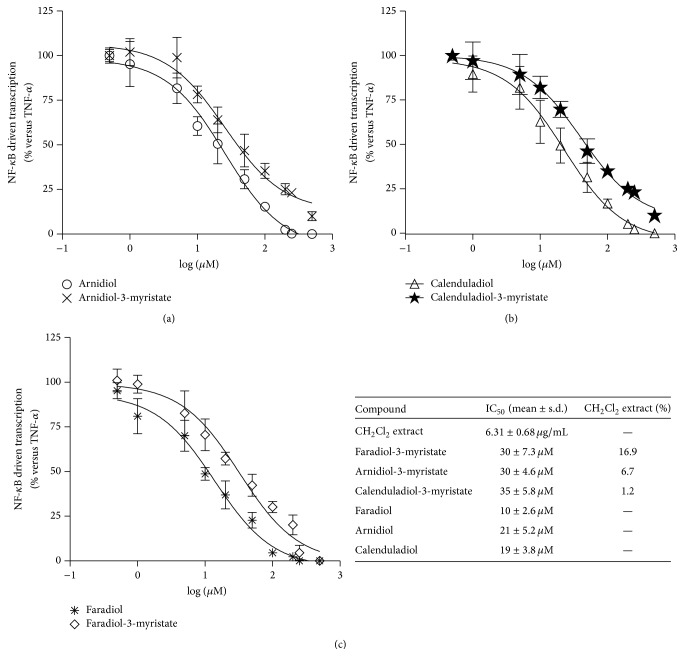
Effect of triterpene esters and the corresponding alcohols ((a) arnidiol-3-myristate, arnidiol; (b) calenduladiol-3-myristate, calenduladiol; (c) faradiol-3-myristate, faradiol) on the NF-*κ*B driven transcription. AGS cells were transiently transfected with NF-*κ*B-luc plasmid and treated with TNF-*α* in the presence of triterpene esters or alcohols (1–50 *μ*M) for 24 h. The table reports the IC_50_ values and relative percentages (w/w) of triterpene esters in lipophilic extract.

For the first time our results demonstrated that all triterpenoid esters exhibited a significant and concentration-dependent inhibitory activity of the NF-*κ*B driven transcription (Figure 2). Triterpene alcohols are not present in CH_2_Cl_2_ extract; however they could be released after ingestion of marigold preparations and are thought to have a greater biological activity; thus we synthesized [27] and assayed them. As expected, triterpene alcohols demonstrated a stronger inhibitory effect on the NF-*κ*B driven transcription than the corresponding esters (Figure 2). Faradiol and faradiol-3-myristate were the most potent compounds (IC_50_  30 ± 7.3 *μ*M and 10 ± 2.6 *μ*M, resp.). However the relative concentrations of these compounds in the extract (Figure 2) revealed that other components contributed to the antiphlogistic effect shown by the extract.

#### 3.2.2. Identification and Biological Activity of Compounds Present in Fraction J

**Figure 3:**
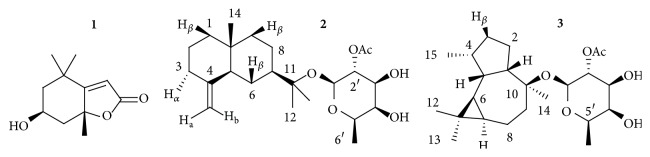
Chemical structure of compounds identified in fraction J.

Fraction J showed the strongest inhibition of the NF-*κ*B driven transcription (IC_50_  4.29 ± 0.97 *μ*g/mL) and its action was comparable to that observed for the lipophilic extract. Thus, J was separated by HPLC on RP-18 column (method 1), into two subfractions: the first contained mainly loliolide (25% w/w), the second (8% w/w) consisted of a mixture of few compounds. Loliolide is a bitter component previously described in* Calendula officinalis* L. flowers [35] and in other plants, including* Fumaria officinalis*, and in different marine algae [36]. It was considered a phytotoxic compound and a biomarker of photooxidative alterations [36]. Recently, it did not exhibit any antiphlogistic effect in a macrophage cell line stimulated with LPS [37]. According to these findings, loliolide revealed no activity on the NF-*κ*B cascade in our cellular model, exhibiting a 10% inhibition on the NF-*κ*B driven transcription at the concentration of 5 *μ*M (corresponding to the IC_50_ of fraction J). The second subfraction inhibited the NF-*κ*B driven transcription in a concentration-dependent manner (IC_50_  3.11 ± 2.30 *μ*g/mL), largely contributing to the effect demonstrated by fraction J. Subsequently, an improved analytical method (method 2) allowed the separation of fraction J into 10 HPLC peaks which corresponded to pure compounds. Those of interest contained the following compounds:** 1** (peak 1, *t*_*R*_ = 2.1 min, mg 8.9);** 2** (peak 4, *t*_*R*_ = 17.2 min, mg 3.4);** 3** (peak 6, *t*_*R*_ = 19.6 min, mg 2.7). The compounds** 2** and** 3** were peracetylated and the products were analyzed by EI-MS without any preliminary purification [28]. The examination of 1D- and 2D-NMR spectra as well as the MS data of those compounds allowed their structural characterization: loliolide** 1**, *β*-eudesmol 11-*O*-*β*-D-(2′-acetyl)-fucopyranoside** 2**, and viridiflorol 10-*O*-*β*-D-(2′-acetyl)-fucopyranoside** 3** (Figure 3). The spectroscopic data of loliolide were identical with those from the literature [38]. This is the first report on the isolation and structural elucidation of the natural product** 2**; spectral data of compound** 3** has been previously reported [39] but the authors assigned the opposite configuration at C-10.

Compound** 2**: C_23_H_38_O_6_, white, amorphous powder; [*α*]^25^_D_ +10.0 (*c* 0.4, CHCl_3_); ESI-MS (positive mode):* m*/*z* 433 [M+Na]^+^; MS^2^ of [M+Na]^+^ *m/z* 229 [M+Na-C_15_H_24_]^+^, MS^3^ of [M+Na]^+^ → [M+Na-C_15_H_24_]^+^ *m/z* 211 [M+Na-C_15_H_24_-H_2_O]^+^; EIMS:* m*/*z* 273 [M-C_15_H_25_O]^+^ (28), 204 [M-C_12_H_18_O_8_]^+•^ (20), 43 (100). HREIMS:* m*/*z* 273.0973 (calcd. for C_12_H_17_O_7_, 273.0974), 204.1878 (calcd. for C_15_H_24_, 204.1878). ^1^H NMR (CDCl_3_, 400 MHz)  *δ*: ~1.49 (1H,* m*, H-1*β*), 1.16 (1H,* dd*, H-1*α*), ~1.59 (2H,* m*, H-2), 2.29 (1H,* br.d*, H-3*β*), 1.98 (1H,* br.q*, H-3*α*), 1.72 (1H,* br.d*, *J* = 12.0 Hz, H-5), 1.66 (1H,* br.d*, *J* = 12.7 Hz, H-6*α*), 1.09 (1H,* q*, *J* = 12.0 Hz, H-6*β*), 1.44 (1H,* tt*, H-7), ~1.57 (1H,* m*, H-8*α*), ~1.23 (1H,* m*, H-8*β*), 1.42 (1H,* br.d*, H-9*β*), ~1.25 (1H,* m*, H-9*α*), 1.17 (3H,* s*, H-12), 1.21 (3H,* s*, H-13), 0.68 (3H,* s*, H-14), 4.70 (1H,* br.s*, H-15a), 4.42 (1H,* br.s*, H-15b), 4.54 (1H,* d*, *J* = 7.9 Hz, H-1′), 4.82 (1H,* dd*, *J* = 9.7, 7.9 Hz, H-2′), 3.62 (1H,* br.d*, H-3′), 3.69 (1H,* br.d*, *J* = 3.6 Hz, H-4′), 3.61 (1H,* br.q*, H-5′), 1.32 (3H,* d*, *J* = 6.6 Hz, H-6′), 2.10 (3H,* s*, H-2′′); ^13^C NMR (CDCl_3_, 100 MHz) *δ*: 41.4 (*t*, C-1), 23.5 (*t*, C-2), 36.9 (*t*, C-3), 150.8 (*s*, C-4), 49.8 (*d*, C-5), 24.6 (*t*, C-6), 48.4 (*d*, C-7), 22.2 (*t*, C-8), 41.9 (*t*, C-9), 36.0 (*s*, C-10), 80.1 (*s*, C-11), 22.8 (*q*, C-12), 25.0 (*q*, C-13), 16.4 (*q*, C-14), 105.3 (*t*, C-15), 95.0 (*d*, C-1′), 73.5 (*d*, C-2′), 73.9 (*d*, C-3′), 72.1 (*d*, C-4′), 69.9 (*d*, C-5′), 16.5 (*q*, C-6′), 171.2 (*s*, C-1′′), 21.0 (*q*, C-2′′); NOESY: 14↔2 and 6*β* and 8*β*; 3*β*  ↔  15a; 15b  ↔  6*α* and 6*β*; 1′  ↔  12 and 13. HMBC: (1) → 14; (10) → 14; (5) → 14; (7) → 12 and 13; (11) → 1′ and 12 and 13; (1′′) → 2′′.

Compound** 3**: C_23_H_38_O_6_, white, amorphous powder; [*α*]^25^_D_ –6.8 (*c* 0.15, CHCl_3_); ESI-MS (positive mode):* m*/*z* 433 [M+Na]^+^; MS^2^ of [M+Na]^+^:* m/z* 229 [M+Na-C_15_H_24_]^+^. EIMS:* m*/*z* 494 [M]^+•^ (0.3), 273 [M-C_15_H_25_O]^+^ (16), 204 [M-C_12_H_18_O_8_]^+•^ (71), 43 (100). HREIMS:* m*/*z* 494.2870 (calcd. for C_27_H_42_O_8_, 494.2880), 273.0975 (calcd. for C_12_H_17_O_7_, 273.0974), 204.1871 (calcd. for C_15_H_24_, 204.1878). ^1^H NMR (CDCl_3_, 400 MHz) *δ*: ~1.88 (1H,* m*, H-1), ~1.60 (2H,* m*, H-2), ~1.81 (1H,* m*, H-3*β*), ~1.28 (1H,* m*, H-3b), ~1.92 (1H,* m*, H-4), 1.70 (1H,* m*, H-5), 0.07 (1H,* t*, *J* = 9.5 Hz, H-6), 0.58 (1H,* td*, *J* = 9.5, 9.5, 7.9 Hz, H-7), ~1.53 (2H,* m*, H-8), ~1.76 (1H,* m*, H-9*β*), ~1.57 (1H,* m*, H-9b), 0.95 (3H,* s*, H-12), 1.00 (3H,* s*, H-13), 1.17 (3H,* s*, H-14), 0.92 (3H,* d*, *J* = 6.7 Hz, H-15), 4.58 (1H,* d*, *J* = 7.9 Hz, H-1′), 4.85 (1H,* dd*, *J* = 7.9, 9.5 Hz, H-2′), 3.59 (1H,* m*, H-3′), 3.67 (1H,* br.d*, *J* = 4 Hz, H-4′), 3.58 (1H,* qd*, *J* = 6.5, 1.0 Hz, H-5′), 1.30 (3H,* d*, *J* = 6.5 Hz, H-6′), 2.09 (3H,* s*, H-2′′); ^1^H NMR (CD_3_OD, 400 MHz) *δ*: ~1.94 (1H,* m*, H-1), 1.71–1.55 (2H,* m*, H-2), ~1.83 (1H,* m*, H-3a), ~1.28 (1H,* m*, H-3b), ~1.96 (1H,* m*, H-4), ~1.73 (1H,* m*, H-5), 1.23 (1H,* t*, H-6), 0.60 (1H,* ddd*, H-7), 1.59 (H,* m*, H-8a), ~1.47 (H,* m*, H-8b), ~1.74 (1H,* m*, H-9a), ~1.62 (1H,* m*, H-9b), 0.97 (3H,* s*, H-12), 1.01 (3H,* s*, H-13), 1.18 (3H,* s*, H-14), 0.94 (3H,* d*, H-15), 4.63 (1H,* d*, H-1′), 4.96 (1H,* dd*, H-2′), 3.64–3.60 (3H,* m*, H-3′ and H-4′ and H-5′), 1.23 (3H,* d*, H-6′), 2.05 (1H,* m*, H-2′′); ^13^C NMR (CDCl_3_, 100 MHz) *δ*: 54.2 (*d*, C-1), 25.4 (*t*, C-2), 28.9 (*t*, C-3), 38.4 (*d*, C-4), 39.7 (*d*, C-5), 22.1 (*d*, C-6), 28.7 (*d*, C-7), 18.2 (*t*, C-8), 37.9 (*t*, C-9), 82.0 (*s*, C-10), 18.7 (*s*, C-11), 16.1 (*q*, C-12), 28.6 (*q*, C-13), 26.6 (*q*, C-14), 16.2 (*q*, C-15), 94.5 (*d*, C-1′), 73.7 (*d*, C-2′), 73.9 (*d*, C-3′), 72.2 (*d*, C-4′), 69.8 (*d*, C-5′), 16.5 (*q*, C-6′), 171.1 (*s*, C-1′′), 21.0 (*q*, C-2′′); NOESY: 6 ↔ 3b and 7 and 13 and 15; 5 ↔ 12; 1′  ↔  1 and 14; 2′′  ↔  12. HMBC: (1) → 14; (9) → 14; (10) → 14 and 1′; (6) → 12 and 13; (7) → 12 and 13; (11) → 12 and 13; (1′′) → 2′ and 2′′.

**Figure 4:**
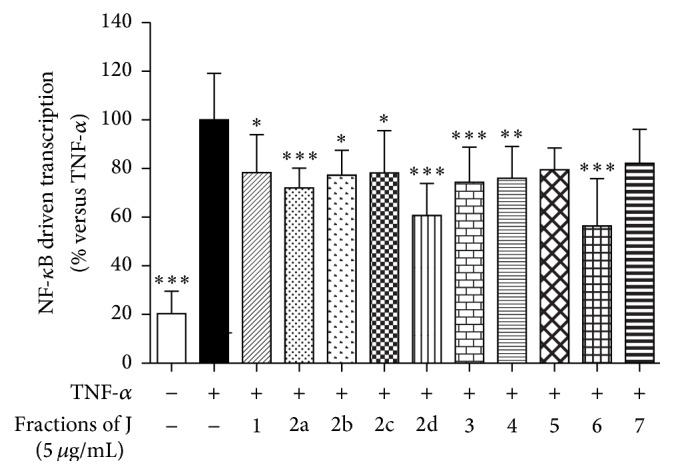
Effect of fractions J.1–J.7 on the NF-*κ*B driven transcription. AGS cells were transiently transfected with NF-*κ*B-luc plasmid and treated with TNF-*α* in the presence of fractions (5 *μ*g/mL) for 24 h. The NF-*κ*B driven transcription was proportional to luciferase signal measured. ^*∗*^ *P* < 0.05, ^*∗∗*^ *P* < 0.01, and ^*∗∗∗*^ *P* < 0.001.

We assayed the 10 peaks for their biological activity on AGS cells. All compounds exhibited a mild inhibitory activity on the NF-*κ*B driven transcription (Figure 4), thus pointing out that together they participate in the biological effect of fraction J. We identified peak 1 as loliolide and peaks 4 and 6 as the fucoside acetates of *β*-eudesmol and viridiflorol, respectively. Similar glycosides were previously described as components of* Calendula persica* C. Mey [40] and many additional analogues have been recently characterized in* Calendula officinalis* L. flowers [28]. It has been shown that *β*-eudesmol exerts an anti-inflammatory activity* in vitro* [41, 42] through inhibition of the NF-*κ*B driven transcription and activation at concentrations (*μ*molar order) similar to that occurring in fraction J [42]. These evidences are in agreement with our results obtained on fraction J and led to the hypothesis that the presence of derivatives of *β*-eudesmol might explain the inhibitory effect of fraction J on the NF-*κ*B activation.

## 4. Conclusions

The present study provides some experimental evidence that lipophilic preparations from* Calendula officinalis* L. may exert an anti-inflammatory activity on the gastric district through the inhibition of the NF-*κ*B system. Results obtained through a bio-guided fractionation of a lipophilic extract from marigold flowers show for the first time that the inhibitory effect demonstrated by the extract on the NF-*κ*B activation could be ascribed to the presence of a mixture of components (the so-called phytocomplex) among which triterpene esters and derivatives of *β*-eudesmol play a pivotal role.

The results described in this work could be of great interest for the following reasons: (1) in the present study, the anti-inflammatory activity of a lipophilic extract from marigold flowers has been proven for internal use. Despite the toxicity renowned for this type of extract, now, in the literature, toxic events after oral administration of marigold preparations are not reported; (2) even if other* in vivo* studies are needed to confirm the antiphlogistic activity and the safety of this type of extract, the identification of some biologically active compounds could be the first step for the formulation of preparations enriched with individual components or extracts from marigold (as faradiol esters or *β*-eudesmol derivatives) useful in the treatment or prevention of gastric inflammatory diseases.

## Figures and Tables

**Table 1:** Bio-guided fractionation of CH_2_Cl_2_ extract and IC_50_ of fractions on the NF-*κ*B driven transcription. AGS cells were transiently transfected with NF-*κ*B-luc plasmid and treated with TNF-*α* in the presence of fractions (2.5–20 *μ*g/mL) for 24 h. NF-*κ*B driven transcription was proportional to luciferase signal measured. The inhibitory activity of fractions was considered significant if ≤50 *μ*g/mL.

Fraction	Hexane : AcOEt	IC_50_ (mean ± s.d.)
A	95 : 5	≥50 *μ*g/mL
B	90 : 10	≥50 *μ*g/mL
C	80 : 20	≥50 *μ*g/mL
D	70 : 30	23.01 ± 8.51 *μ*g/mL
E	60 : 40	≥50 *μ*g/mL
F	50 : 50	≥50 *μ*g/mL
G	40 : 60	≥50 *μ*g/mL
H	30 : 70	TOXIC
I	20 : 80	TOXIC
J	10 : 90	4.29 ± 0.97 *μ*g/mL
K	AcOEt	19.59 ± 5.77 *μ*g/mL
L	AcOEt	15.52 ± 5.21 *μ*g/mL
M	Acetone	12.84 ± 4.43 *μ*g/mL
N	Acetone	11.59 ± 3.26 *μ*g/mL
O	Act∖iPrOH 9 : 1	7.82 ± 2.13 *μ*g/mL
P	Act∖iPrOH 9 : 1	≥50 *μ*g/mL
